# Neuroendoscopic minimally invasive surgery and small bone window craniotomy hematoma clearance in the treatment of hypertensive cerebral hemorrhage

**DOI:** 10.12669/pjms.35.2.463

**Published:** 2019

**Authors:** Chengjia Gui, Yikuan Gao, Dan Hu, Xinyu Yang

**Affiliations:** 1 *Chengjia Gui, Department of Neurosurgery, Tianjin Medical University General Hospital, Tianjin 300052, China; Department of Neurosurgery, Central Hospital of Yongzhou, Hunan, 425000, China*; 2 *Yikuan Gao, Department of Neurosurgery, Tianjin Medical University General Hospital, Tianjin 300052, China; Department of Neurosurgery, Central Hospital of Yongzhou, Hunan, 425000, China*; 3 *Dan Hu, Department of Neurosurgery, Central Hospital of Yongzhou, Hunan, 425000, China*; 4 *Xinyu Yang, Department of Neurosurgery, Tianjin Medical University General Hospital, Tianjin 300052, China*

**Keywords:** Hypertension, Cerebral hemorrhage, Neuroendoscopy, Small bone window craniotomy

## Abstract

**Objective:**

To analyze the effects of neuroendoscopic minimally invasive surgery and small bone window craniotomy hematoma clearance through comparing clinical indicators of the two operation modes and to provide a reference for selection of proper minimally invasive surgery.

**Methods:**

One hundred and twenty-six patients with hypertensive cerebral hemorrhage who received diagnosis and treatment in our hospital between December 2015 and December 2017 were selected and grouped into an observation group (n=63) and a control group (n=63) using random number table. Patients in the observation group were treated by neuroendoscopic surgery, while patients in the control group were treated by small bone window craniotomy. The surgical condition, clinical effect and prognosis of the two groups were analyzed and compared.

**Results:**

Patients in the observation group completed surgery in a shorter time and bled less during operation compared to the control group, and the hematoma clearance rate of the observation group was obviously higher than that of the control group; the differences had statistical significance (P<0.05). The nerve deficiency scale (NDS) scores of the two groups at the postoperative 3^rd^ month were lower than those before surgery (P<0.05), and the activity of daily life (ADL) score at the postoperative 3^rd^ month was higher than that before surgery (P<0.05). The observation group had lower NDS score and higher ADL score compared to the control group, and the differences had statistical significance (P<0.05). The incidence of complications of the observation group was lower than that of the control group after surgery, and the rate of favourable prognosis of the observation group was higher than that of the control group at the postoperative 3^rd^ month (P<0.05).

**Conclusion:**

Neuroendoscopic surgery is more effective and safe, causes less bleeding and has better prognosis and nerve function recovery compared to small bone window craniotomy in the treatment of hypertensive cerebral hemorrhage.

## INTRODUCTION

Hypertensive intracerebral hemorrhage (HICH) is one of the common acute cerebrovascular diseases and also one of the common types of cerebral hemorrhage. Now it has been an important cause of death,^1^ which has severely threatened the physical and psychological health of human. Guidelines for the Management of Spontaneous Intracerebral Hemorrhage released by American Heart Association in 2015 pointed out that about 10~15% of patients who were attacked by stroke for the first time had HICH, the mortality rate was about 50%, about half of the dead died within 24 hours after onset,^2^ and only 28%~35% of the survivors had independent living ability.^3^ Someone in China found that the incidence of HICH in China was 0.72/10000~1.15/10000, most of them aged over 55 years, and the incidence among young people became increasingly higher.^4^,^5^ There are many patients with HICH in China because of the large population base, which brings huge burdens to the family members of the patients and the society and severely affects the living level of people. Therefore how to select effective diagnostic and treatment methods for HICH, improve the survival rate and enhance living quality of patients in the late stage has been the dilemma.

The key of treating HICH is eliminating space occupying effect, removing hematoma, decreasing intracranial pressure, repairing injured neurons, preventing secondary pathological injury and promoting prognosis as soon as possible.^6^ Patients with HICH who have a small amount of blood loss can get a good outcome after being treated by drugs. Patients who bleed more than 30 mL are usually treated by surgery.^7^ Gregson et al. found that surgical treatment was more effective than conservative treatment for patients with cerebral hemorrhage who underwent early surgery within eight hours after hemorrhage,^8^ had 20~50 mL of blood loss, had 9~12 points of Glasgow Coma Scale (GCS) score, and aged 50~69 years. Small bone window craniotomy is a traditional surgical method for treating HICH, and its effect has been widely recognized. But it has shortcomings of large trauma and multiple postoperative complications.^9^,^10^ With the development of minimally invasive technology, endoscopic neurosurgery has been extensively performed in clinics for its advantages of less pain, fast recovery and low incidence of postoperative complications. It has been the main surgical method for treating HICH.^11^,^12^

Through comparing the effects of endoscopic neurosurgery, stereotactic treatment and craniotomy for 30 patients with cerebral hemorrhage in basal ganglia, Cho et al. found that endoscopic neurosurgery had the highest hematoma clearance rate and the shortest operation time.^13^ However, the treatment criteria have not been unified though there are many studies concerning neuro endoscopy. The selection of surgical method, therapeutic effect and prognosis of HICH are still being explored. In this study, the effects of neuro endoscopic minimally invasive surgery and small bone window craniotomy were compared. This study can guide clinical physicians to select reasonable treatment schemes for HICH and figure out the effectiveness of endoscopic neurosurgery in the treatment of HICH.

## METHODS

One hundred and twenty-six patients with HICH who received diagnosis and treatment in our hospital between December 2015 and December 2017 were enrolled. All of them satisfied the diagnostic criteria formulated by WHO/ISH in 1999.^14^ They were admitted to the hospital within 12 hours after onset and were definitely diagnosed by head computed tomography (CT). The patients had 30~60 mL of supratentorial hemorrhage, 5~13 points of GCS score, grade II~IV consciousness disorder, and history of hypertension. Patients who had more than 60 mL of blood loss, need undergoing decompressive craniectomy because of severe cerebral edema, had cerebellar hemorrhage, brainstem hemorrhage, craniocerebral injury, arterial aneurysm or arteriovenous malformation, or have took anticoagulant drugs for a long term were excluded. The patients were divided into an observation group and a control group using random number table, 63 cases in each group. In the observation group, there were 39 males and 24 females, and they aged 40~71 years (54.02±3.74 years) and had a hypertension history of 4~21 years (average 8.21±1.06 years), 35~73 mL of hematoma (average 52.39±3.64) and 4~12 points of GCS score (average 6.01±0.64 points). In the control group, there were 36 males and 27 females, and they aged 37~71 years (average 52.33±3.41 years) and had a hypertension history of 3~22 years (average 7.38±0.75 years), 30~69 mL of hematoma and 3~13 points of GCS score (average 6.35±0.71 points). The differences of gender and age between the two groups had no statistical significance (P>0.05); hence the results were comparable. This study has been approved by the ethical committee of our hospital, and all the patients signed informed consent.

Patients in the observation group underwent endoscopic neurosurgery. The position of hematoma was determined using head CT. The site which was the nearest to the largest hematoma and away from important nerves, blood vessels and brain function region was taken as the surgical approach. After general anesthesia, a 5 cm incision was made. The scalp tissue was retracted to fully expose the cranium. A bone hole whose diameter was 2 cm was drilled. The endocranium was incised. A neuroendoscopy set (STORZ, Germany) was punctured into the hematoma cavity. After the inner core was removed, the neuroendoscope was inserted. The hematoma was removed under the neuro endoscopy. Electrocoagulation could be used if there was bleeding. Drainage tubes were inserted after surgery. Finally the incision was sutured.

Patients in the control group underwent small bone window craniotomy. After general anesthesia, the position of hematoma was determined according to the results of head CT. A 6cm incision was cut on the scalp. Then a hole was drilled. The size of small bone window was about 4.0 cm×5.0 cm. The endocranium was incised. Then the position of hematoma was determined using brain puncture. The cerebral cortex was incised. After hematoma elimination, epidural drainage tube was inserted. Then the cranium was closed. After surgery, patients in both groups were given treatment to resist infection, maintain water and electrolytes balance and reduce intracranial pressure.

The intraoperative blood loss, duration of surgery, hematoma clearance rate and incidence of postoperative complications were compared and analyzed. The formula of hematoma clearance rate was: hematoma clearance rate = (preoperative hematoma volume under CT - hematoma volume under CT within 24 hour after surgery)/preoperative hematoma volume under CT. All the patients were followed up for three months. The degree of neurological deficit of the patients was evaluated using clinical nerve deficiency scale (NDS) before surgery and at the postoperative 3^rd^ month, and the score was 0 point ~ 45 points. The high the NDS score, the severer the neurologic impairment. The ability of daily living was evaluated using activity of daily living scale (ADL) before surgery and at the postoperative 3^rd^ month. Higher score of ADL indicated better activity of daily living. Good recovery was given five points; mild disability was given four points; severe disability was given 3 points; vegetative state was given two points; death was given one point. Favourable prognosis was given 4 or 5 points, and poor prognosis was given 1~3 points.

### Statistical method

Data were processed using SPSS ver. 21.0. Measurement data were expressed as mean±standard deviation (SD) and tested by t test. Categorical data were expressed as rate (%) and tested by Chi-square test. Difference was considered statistically significant if P<0.05.

## RESULTS

The duration of surgery and intraoperative blood loss of the observation group were less than those of the control group, and the hematoma clearance rate of the observation group was obviously higher than that of the control group (P<0.05, Table I).

**Table-I:** Surgical indexes between the two groups (mean±SD).

Group	Duration of surgery (h)	Intraoperative blood loss (mL)	Hematoma clearance rate (%)
Observation group	1.69±0.87	35.61±13.52	92.31±8.52
Control group	3.69±1.34	277.14±101.33	78.23±7.74
t	8.497	23.269	8.574
p	<0.05	<0.05	<0.05

The differences of ADL and NDS scores between the two groups had statistical significance before treatment (P>0.05). The NDS score of the two groups at the postoperative 3^rd^ month was lower than that before treatment, and the ADL score at the postoperative 3^rd^ month was higher than that before treatment (P<0.05). The NDS score of the observation group was obviously lower than that of the control group, and the ADL score of the observation group was obviously higher than that of the control group, as shown in Table II.

**Table-II:** ADL and NDS score between the two groups (mean±SD).

Group	Time	ADL score	NDS score
Observation group	Before treatment	29.87±3.73	41.26±5.67
	Postoperative 3^rd^ month	66.86±5.87^*#^	22.12±3.01^*#^
Control group	Before treatment	30.01±3.82	40.79±5.74
	Postoperative 3^rd^ month	44.79±4.52^*^	31.72±3.19^*^

***Note:***

* indicated P<0.05 compared to before treatment;

# indicated P<0.05 compared to the control group.

In the control group, there were four cases of intracranial infection and five cases of pulmonary infection; the incidence of complications was 14.29%. In the observation group, there was only one case of pulmonary infection; the incidence of complications was 1.59%. The incidence of complications of the observation group was significantly lower than that of the control group (X^2^=4.913, P<0.05).

**Fig.1:**
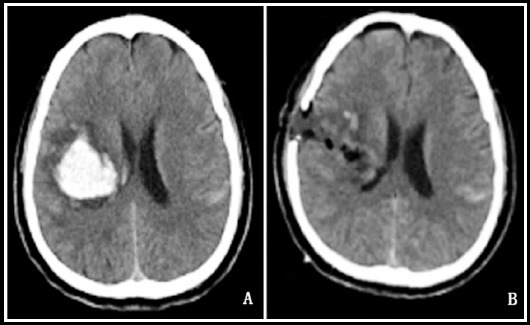
Head CT of a 58-year-old female patient in the observation group before and after surgery (A: preoperative; B: postoperative).

**Fig.2:**
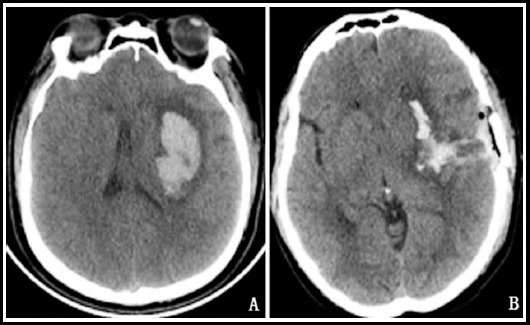
Head CT of a 53-year-old female patient in the control group before and after surgery (A: preoperative; B: postoperative).

The rate of favorable prognosis of the observation group was 61.90% at the postoperative 3^rd^ month, which was significantly higher than that of the control group (41.27%) (X^2^=4.174, P<0.05, Table III). The typical cases are shown in Fig. 1 and 2.

**Table-III:** Comparison of prognosis between the two groups at the postoperative 3^rd^ month [n(%)].

Group	1 point	2 points	3 points	4 points	5 points	Favourable prognosis
Observation group	4(6.35)	7(11.11)	13(20.34)	15(23.81)	24(38.09)	39(61.90)^#^
Control group	9(14.29)	11(17.46)	17(26.98)	12(19.05)	14(22.22)	26(41.27)

***Note:***

# indicated P<0.05 compared to the control group.

## DISCUSSION

HICH refers to a hemorrhagic disease which occurs secondary to hypertension, which has a high incidence in China. HICH can produce primary or secondary damages to brain tissues.^15^ Primary damage mainly manifests as cerebral hernia induced by the compression on brain tissues because of the space occupying effect of hematoma. It may threaten the lives of patients in severe cases. Secondary damage which is induced by the substances released by brain cell damages and hemolysis can further induce edema and dysfunction of brain tissues, aggravating disease condition.^16^ As the occupied effect induced by acute intracranial hematoma and pathological damage induced by bleeding is the main cause of disability and death, intracranial hematoma should be cleaned by clinical treatment as soon as possible to relieve the occupied effect of hematoma, release brain tissue oppressed by hematoma, alleviate encephaledema, reduce intracranial pressure, and improve the prognosis of patients.^2^,^17^

There are many operation methods for treating HICH currently, but the standard method is still controversial, and every surgical procedures has indications and complications. In the application of craniotomy evacuation of hematoma, operation can be performed under direction vision, hematoma can be thoroughly removed, the hemostatic effect is definite. Moreover whether decompressive craniectomy is needed or not can be determined according to intraoperative intracranial pressure. Its complications include large trauma, severe hemorrhage, long duration of surgery and poor prognosis.^18^ Under the influence of the general tendency of minimally invasive surgery, neuroendoscopy has gained substantial development in the field of neurosurgery.^19^ Neuroendoscopy has special advantages in the treatment of HICH, including removal of hematoma under direct vision, multi-angle rotation of endoscope, small trauma, short duration of operation and small blood loss. Under direct vision, the good lighting can help fully expose lesions and reduce damages to neurons and important blood vessels. The multi-angle rotation of endoscope can help thoroughly remove hematoma at different positions. Someone^20^ pointed out that the hematoma clearance rate of endoscopic neurosurgery was between 86% and 100% and that endoscopic neurosurgery had advantages of small trauma, short operation time and small bleeding volume. The results of this study demonstrated that the observation group had shorter operation time, smaller intraoperative bleeding volume and significantly higher hematoma clearance rate compared to the control group, which was consistent with the aforementioned mechanism and previous research results.

The long duration of craniotomy has large damages to the whole body and brain of patients. Therefore surgery associated complications including infection, organ failure, rehaemorrhagia and thrombogenesis are prone to occur in the respiratory system, urinary system, nervous system and digestive system after craniotomy.^21^Though small bone window craniotomy can effectively reduce the incidences of some complications, the incidence of surgery associated complications is still up to 40%, and some patients have died of the complications.^22^ Neuroendoscopic hematoma evacuation can also induce similar complications in the treatment of cerebral hemorrhage, but the incidence of complications is much lower. It is found that the incidence of complications of neuroendoscopic hematoma evacuation was 3.3%, much lower than that of stereotactic intracranial hematoma removal (10%) and craniotomy (16.6%) in the treatment of cerebral hemorrhage in basal ganglia (P<0.05).^13^ The results of this study demonstrated that the incidence of postoperative complications of the observation group was significantly lower than that of the control group, which was consistent with the aforementioned research result.

The neurological recovery, activity of daily living and long-term curative effect of the patients were evaluated using NDS, ADL and GCS scores respectively at the postoperative 3^rd^ month. The results demonstrated that the NDS score of the observation group was significantly lower than that of the control group, the ADL score of the observation group was significantly higher than that of the control group, and the rate of favourable prognosis of the observation group was significantly higher than that of the control group, suggesting that endoscopic neurosurgery had special advantage in improving the neurological function, living ability and prognosis of patients with HICH. The finding was consistent with the research results of Chan et al.^23^ The main reason of the above result is the small trauma that endoscopic neurosurgery brought to patients, not only reflecting on the small damages to brain tissue, but also reflecting on the short duration of surgery, small blood loss and low tolerance to surgery. Therefore the patients recovered fast, had less complication, their hospital stay was short, and started functional exercise earlier.

## CONCLUSION

Endoscopic neurosurgery has advantages of small trauma, mild blood loss, and short duration of surgery, high hematoma clearance rate, low incidence of complications, rapid recovery and favorable prognosis for patients with HICH. With the constant development of science and technology and the accumulation of experience in using neuroendoscopy, neuroendoscopy is bound to play an increasingly important role in the field of neurosurgery and will be accepted by more and more physicians.
